# Development and Validation of a Gas Chromatography–Mass Spectrometry Method for the Analysis of the Novel Plant-Based Substance with Antimicrobial Activity

**DOI:** 10.3390/antibiotics12101558

**Published:** 2023-10-22

**Authors:** Viktor A. Filatov, Egor A. Ilin, Olesya Yu. Kulyak, Elena I. Kalenikova

**Affiliations:** 1 Department of Pharmaceutical Chemistry and Organization of Pharmaceutical Business, Faculty of Basic Medicine, Lomonosov Moscow State University, 119991 Moscow, Russia; kulyak-olesya@mail.ru (O.Y.K.); eikaleni@fbm.msu.ru (E.I.K.); 2 Science Center, SkyLab AG, 1066 Lausanne, Switzerland; 3 Department of Chemistry, Lomonosov Moscow State University, 119991 Moscow, Russia; ilin.eg.2000@gmail.com; 4 N. D. Zelinsky Institute of Organic Chemistry, 119991 Moscow, Russia; 5 All-Russian Scientific Research Institute of Medicinal and Aromatic Plants, 117216 Moscow, Russia

**Keywords:** tea tree essential oil, eucalyptol, bisabolol, terpenes, GC–MS, validation

## Abstract

The research into new pharmaceutical substances based on essential oils, individual biologically active phytochemicals, and plant extracts is a priority in field of pharmaceutical sciences. A novel multicomponent substance based on *Melaleuca alternifolia* (*M. alternifolia*) leaf oil (TTO), 1,8-cineole (eucalyptol), and (-)-α-bisabolol with potent synergetic antimicrobial activity was investigated and suggested for the treatment of seborrheic dermatitis (SD) and dandruff. The objective of this research was to establish and validate a specific, accurate, and precise gas chromatography–mass spectrometry (GC–MS) method for further quantitative and qualitative analysis in order to ensure quality control. The main parameters of validation were suitability, specificity, linearity, accuracy, and intermediate precision according to the *European Pharmacopoeia* (XI edition), *Russian Pharmacopoeia* (XIV edition), and some parameters of ICH requirements. The peaks of fifteen chemical phytoconstituents were identified in the test sample solution with the prevalence of (−)-α-bisabolol (27.67%), 1,8-cineole (25.63%), and terpinen-4-ol (16.98%). These phytochemicals in the novel substance were chosen for standardization and validation of the GC–MS method. The chosen chromatographic conditions were confirmed for testing of the plant-based substance in a suitability test. It was established that the GC–MS method provides a significant separation, symmetry of peaks and resolution between phytochemicals. The calibration curves of each phytochemical had good linearity (R^2^ > 0.999) in five concentrations. In the same concertation range, the accuracy of terpinen-4-ol, 1,8-cineol, and (−)-α-bisabolol determination using the method of additives was 98.3–101.60%; the relative standard deviation (RSD) ranged from 0.89% to 1.51% and corresponded to requirements. The intraday and interday precision was ≤2.56%. Thus, the GC–MS method was validated to be specific, sensitive, linear, accurate, and precise. This GC–MS method could be recommended as a routine analytic technique for multicomponent plant-based substances-enriched terpenes.

## 1. Introduction

The development of novel substances of plant origin is a priority in the pharmaceutical field and phytomedicine [1]. Phytomedicine, such as essential oils, different plant extracts, and individual molecules from medical plants, is helpful for the treatment of different skin and scalp diseases [2,3], such as seborrheic dermatitis (SD) [4]. SD is a widespread disorder of the scalp and facial skin, occurring in young people, the adult population, and newborns worldwide [5]. The pathogenesis of SD is related to changes in the scalp microflora, excessive fungal growth, hormonal changes, oxidative stress, and improper hygiene [6]. The synthetic antiseptics, antimicrobials, topical steroidal hormones, and calcineurin inhibitors are recommended as first-choice treatments of SD [6]. However, synthetic antimicrobials have a nonselective activity on the entire scalp microflora, disturb the normal microflora [7], and determine a high risk of antimicrobial resistance [8,9,10,11]. It was confirmed that *Malassezia* species as a main etiological factor of SD had a decreased sensitivity to antifungal and antimicrobial drugs due to the genetic changes, dysregulation of transporters, and drug efflux [12]. Prolonged and uncontrolled application of drugs with ketoconazole for the treatment of SD leads to decreased efficacy and severe adverse effects [13,14,15]. The novel substance based on *Melaleuca alternifolia* (*M. alternifolia*) leaf oil (TTO), 1,8-cineole (eucalyptol), and (−)-α-bisabolol in a specific mass ratio of 1:1:1 was developed and investigated for the treatment of SD [4]. This targeted substance has a synergetic antimicrobial activity against *Malassezia* species, *Staphylococcus epidermidis*, and *Staphylococcus aureus*, which are related to the exacerbation of SD [4,16,17]. The antimicrobial potential of this combination was comparable to clinically recommended benzalkonium chloride [18,19], climbazole [20,21,22], and ketoconazole [6,23,24,25] without damage of the normal scalp microflora [4,17]. The antimicrobial activity of TTO [26,27], 1,8-cineole [28,29], and (−)-α-bisabolol [30,31] was a necessary basis for further targeted synergetic antimicrobial action of the novel substance including these phytochemicals. Nevertheless, the enriched heterogenous phytochemical composition of this substance makes it difficult to analyze the phytochemicals and provide quality control for this substance by analytical methods without corrections of chromatographic conditions [32]. Some characteristics of essential oils and terpenes, such as a high volatility [33], instability due to oxidation [34], chemical reaction during storage [35], and change in the composition of secondary metabolites [36,37,38], complicate the use only one of these methods, such as thin-layer chromatography (TLC), liquid chromatography (LC), optical rotation, and refractometry [39,40,41]. It is worth mentioning that use of standard chromatographic conditions without modifications for gas chromatography coupled to mass spectrometry (GC–MS) analysis of the novel substance based on *Melaleuca alternifolia* (*M. alternifolia*) leaf oil (TTO), 1,8-cineole (eucalyptol), and (−)-α-bisabolol could not provide the sufficient resolution between phytochemicals [4] because of a unique chemical profile defined by their individual phytochemicals. To determine the 1,8-cineole, terpinen-4-ol, and (−)-α-bisabolol in the substance and achieve a proper separation of the phytochemicals, it is necessary to suggest a modification in GC–MS assay parameters, such as a length of column, a column phase, temperature gradient [32], and validate theirs.

The application of modern approach, such as volatilomics, improves the quantitative and qualitative analysis of multicomponent substances-enriched volatile organic compounds due to their volatility and lipophilic properties [33]. This methodology possesses the ability to identify many phytochemicals present in one substance. In the last decades, the GC–MS method as a part of volatilomics approach [33] was suggested for the analysis of lipophilic plant-based substances, such as essential oils, and supercritical plant extracts [33,41]. The GC–MS method is the first-choice analytical technique of the volatilomics approach because of high selectivity, accuracy, and reproducibility [33]. Furthermore, compared to TLC, the GC–MS is more sensitive, precise, accurate, and efficient and could be used to analyze substances of plant origin with the complex chemical composition [38,39,40]. Mass spectrometry (MS) combined with gas chromatography (GC) could identify the chemical structures of terpenes in essential oils, determine the relative content of phytochemicals, and find unknown compounds or impurities [42]. Consequently, the GC–MS method is suitable for qualitative screening and quantitative analysis of multicomponent substances based on essential oils and their phytochemicals [42]. However, selecting the proper chromatographic conditions must be chosen for time saving as well as ensuring an increase in sensitivity, high resistance to matrix interference, and significant resolution of terpenes due to the mass spectral similarity of their isomers in the novel plant-based substance [4,42,43]. The modifications of chromatographic conditions and additional techniques have not been previously developed and validated due to the innovative nature of using this substance for the treatment of SD.

Thus, this study aimed to establish the proper chromatographic conditions and to validate the suggested GC–MS assay. This method could provide a proper quality control of the multicomponent substance and the accompanying drugs. Furthermore, the validated method could contribute to the future implementation of phytomedicines into pharmaceutical products and provide a fundamental basis for the development of modified GC–MS conditions for the analysis of plant-based pharmaceutical substances.

## 2. Results and Discussion

### 2.1. GC–MS Analysis of the Plant-Based Substance

**Figure 1:**
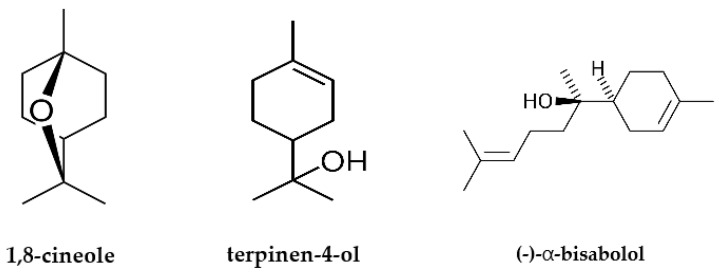
Chemical structures of the main phytoconstituents using GC–MS analysis.

The chemical composition of the standard sample of the plant-based substance based on terpinen-4-ol, 1,8-cineole and (−)-α-bisabolol at a mass ratio 1:1:1 was prepared and analyzed by the GC–MS method (Supplementary Figure S1). The most abundant phytochemicals were 1,8-cineole (42.06%), (−)-α-bisabolol (31.70%), terpinen-4-ol (25.00%), 2-hexanol (0.48%), m-cymene (0.34%), borneol (0.19%), trans-ascaridol glycol (0.17%), and α-bisabolene (0.07%). The identified compounds belonged to the terpenes of essential oils. The peaks of residual organic impurities were not numerous and did not exceed 0.5%. The main phytoconstituents for the standardization of this novel substance and further validation were 1,8-cineole, (−)-α-bisabolol, and terpinen-4-ol, representing 98.76% of the total composition (Figure 1). These compounds had a characteristic retention time (RT), retention indexes (RIs), peaks, and mass spectra (Table 1) that were compared for obtained values and data from the literature in the National Institute of Standards and Technology-2017 (NIST-2017) and Wiley-08 libraries. Quantitative analysis of these three compounds in the novel plant-based substance was carried out further for validation of the GC–MS method that is suitable for the investigation of multicomponent phytomedicines, extracts, essential oils, and their combinations.

Additionally, the test sample of TTO, 1,8-cineole:(−)-α-bisabolol in a 1:1:1 mass ratio was analyzed by the GC–MS method to compare with the standard solution. Fifteen phytochemicals were observed with characteristic RT, *m*/*z* ratio, and peak area (Table 2). The most abundant phytochemicals were 1,8-cineole (25.63%), (−)-α-bisabolol (27.67%), terpinen-4-ol (16.98%), and α-terpineol (15.31%). Other compounds were previously identified in TTO [4]. β-Bisabolol, farnesol, and trans-geranylgeraniol were obtained from a (−)-α-bisabolol substance. However, a high resolution between peaks was achieved due to the chosen conditions of GC–MS assay. The use of MS as a detector was helpful in distinguishing α-phellandrene and α-terpinene between each other (Table 2). Based on this study, 1,8-cineole, terpinen-4-ol, and (−)-α-bisabolol were chosen as the main phytochemicals in the novel plant-based substance for further standardization and validation of the GC–MS method.

Terpinen-4-ol is a specific major compound of TTO [4] that is standardized for terpinen-4-ol content, according to ISO 4730 [45]. The natural TTO must contain at least 30% of terpinen-4-ol in the composition [45]. Terpinen-4-ol has a moderate antimicrobial activity [46] that could be useful for the treatment of skin disorders and SD [4] especially.

1,8-cineole is a major component of essential oils from the *Eucalyptus* species [47,48]. Moreover, 1,8-cineole is a secondary phytochemical metabolite of TTO in an amount up to 10% [49]. This phytochemical has a mild antimicrobial activity [50] and works via enhancing the penetration of different molecules into bacterial and fungal cells [50]. The chemical structure and lipophilicity of 1,8-cineole determines the ability to increase the membrane permeability, change the enzyme function in microbial cells, and consequently suppress the growth of bacteria and fungi [51,52]. Combinations of essential oils or substances with 1,8-cineole revealed an improved antimicrobial activity and decreased the minimal inhibitory concentration (MICs) of compounds together [50] against Gram-positive and Gram-negative bacteria. Additionally, 1,8-cineole can suppress the biofilm formation of pathogenic bacteria [53]. According to GC–MS analysis, the peak area of 1,8-cineole was summarized by presence in TTO and the addition of pure 1,8-cineole to the novel substance during the preparation of test sample solutions for research.

(−)-α-Bisabolol is a famous sesquiterpene from several essential oils with soothing, anti-inflammatory, antiallergic, and antibacterial effects on skin and scalp [54,55]. This compound has helpful properties for the treatment of SD [4], such as suppression of the release of pro-inflammatory cytokines [56], antifungal activity [57], wound healing, and an antinociceptive effect [56]. Moreover, this compound works as an enhancer for the deep delivery of the lipophilic substances into the skin derma [58].

The MS detector is the most recommended detection technique for analysis of volatile, lipophilic, and multicomponent substances of plant origin [59]. This technique provides data about molecular masses of the analyzed phytochemicals and fragment ions and is used for their qualitive and quantitative identification. GC–MS analysis was performed for the full investigation of the novel plant-based substance and evaluation of trace impurities [4]. Experimental results of mass spectrometry (Supplementary Figures S2–S4) are compared with *m*/*z* peaks in the NIST-2017 and Wiley-08 libraries [44,60,61], providing the required analytic parameters for specificity, sensitivity, accuracy, and precision of validated GC–MS assay. The main characteristics of MS for the substance based on TTO, 1,8-cineole, and (-)-α-bisabolol are shown in Table 3.

According to the GC–MS results of the standard sample solution based on 1,8-cineole, terpinen-4-ol, and (−)-α-bisabolol, the five trace phytochemicals were determined. The m-cymene was discovered in TTO in the previous research of TTO [4]. The 2-hexanol [62], borneol [63], trans-ascaridol glycol [32], and α-bisabolene [64] were identified for the first time in the novel plant-based substance. Regarding the new terpenes in the substance, the chemical structure is similar to that of the 1,8-cineole, terpinen-4-ol, and (−)-α-bisabolol and could possibly be generated from 1-methyl-4-(1-methylethenyl)-2-cyclohexene-1-ol, and TTO terpenes [4]. These phytochemicals have an antimicrobial activity [64,65,66] against pathogens involved in the pathogenesis of SD and dandruff. Additional anti-inflammatory activity of the trans-ascaridol glycol [63] improves the scalp condition and decreases the pain sensitivity, itching, severe irritation.

According to the GC–MS results of the standard sample solution based on 1,8-cineole, TTO, and (−)-α-bisabolol, the fifteen phytochemicals were determined. The β-myrcene, α-phellandrene, α-terpinene, D-limonene, γ-terpinene, α-terpineol, δ-cadinene, β-bisabolol, β-caryophyllene, and farnesol were identified in the previous research into the novel plant-based substance with targeted antimicrobial activity against *S. epidermidis*, *S. aureus*, *M. furfur*, and *C. albicans* [4]. Trans-geranylgeraniol was especially found for the first time. This compound in combination with other essential oils or terpenes demonstrated the antimicrobial activity against Gram-positive cocci [67] involved in the pathogenesis of SD. These identified terpenes can decrease the inflammatory reaction, suppress the pain sensitivity, and modulate the immune response [68,69,70]. Overall, the enriched phytochemical composition of the novel substance is responsible for the synergetic antibacterial and antifungal activities against SD-related pathogens [4]. Even minor trace impurities could help in the deep delivery of the terpinen-4-ol, 1,8-cineole, and (−)-α-bisabolol into bacterial and fungal cells. The validation of the GC–MS analysis with proper chromatographic conditions is needed for quantitative screening and qualitative assessment due to the rich chemical composition and reaction ability between compounds during storage.

### 2.2. Validation of GC–MS Analysis

The GC–MS analysis was tested and validated with the use of standard and test sample solutions in accordance with the *European Pharmacopoeia* (XI edition) [71], *Russian Pharmacopoeia* (XIV edition) [72], and some parameters of ICH requirements, such as system suitability, specificity, linearity, accuracy and precision [73,74,75]. The chosen chromatographic system was sensitive, specific, linear, accurate, and reproducible after completing the validation process in terms of suitability, specificity, linearity, accuracy, and reproducibility. The summary of the GC–MS method validation results is shown in Table 4.

#### 2.2.1. Suitability of the Chromatographic System

System suitability is needed to prove that the chromatographic system works perfectly before analysis using equipment with a selected chromatographic condition [74]. *Essential oils* and phytochemicals can influence the chromatographic column and the separation of compounds during analysis [75]; therefore, the *European Pharmacopoeia* (XI edition) [71] and the *Russian Pharmacopoeia* (XIV edition) [72] recommend performing this test. The test was carried out using chromatography of a standard sample of linalyl acetate at 130 °C. The presence of the linalyl acetate and residual organic impurity was illustrated in the resulting chromatogram (Supplementary Figure S5). The peak intensity of the linalyl acetate and organic impurity of linalool corresponded to 99.55% and 0.45%, respectively. It was no observed any destruction of linalyl acetate during GC–MS analysis. The results of this test confirmed the suitability of the selected chromatographic column for GC–MS analysis of the novel substance based on TTO, 1,8-cineole, and (−)-α-bisabolol.

Additionally, the suitability of the selected chromatographic conditions was confirmed via measurement of the main parameters: NTPs, resolution between peaks, and relative standard deviation (RSD) of peaks. The analysis of the standard sample of terpinene-4-ol, 1,8-cineole (−)-α-bisabolol in a 1:1:1 mass ratio confirmed the sufficient efficiency to separate the phytochemicals during GC–MS analysis (Table 5). The NTPs were more than 10,000 theoretical plates. The resolution between terpinen-4-ol, 1,8-cineol, and (−)-α-bisabolol was from 1.5 to 2.0, with a symmetry of peaks. The RSD of peak areas did not exceed 3.0%. These results correspond to pharmacopeial requirements of the acceptance criterion for method suitability.

#### 2.2.2. Specificity

**Figure 2:**
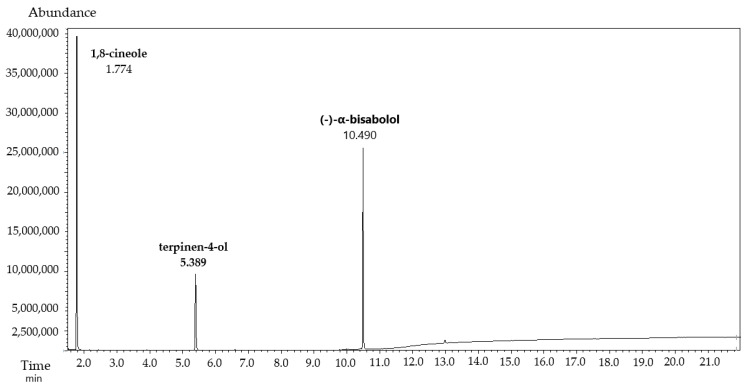
GC–MS chromatogram of the standard solution of the novel plant-based substance (TTO:1,8-cineole:(−)-α-bisabolol in a 1:1:1 mass ratio) in specificity test.

**Figure 3:**
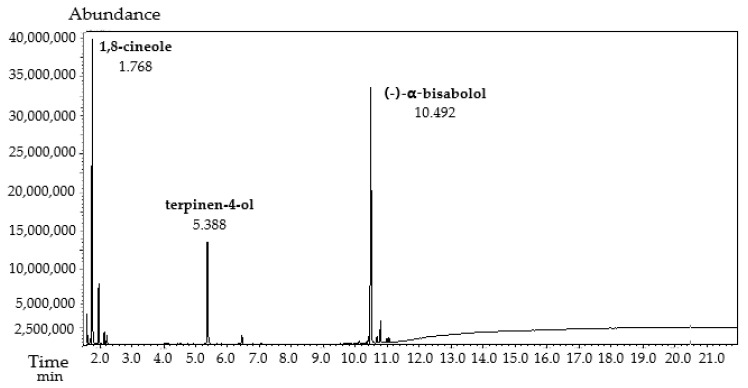
GC–MS chromatogram of the test solution of the novel plant-based substance (terpinen-4-ol:1,8-cineole:(−)-α-bisabolol in a 1:1:1 mass ratio) in specificity test.

The specificity of the GC–MS method was firstly determined to find the main phytochemicals in the novel substance based on 1,8-cineole, TTO-enriched terpinen-4-ol, and (−)-α-bisabolol in a specific mass ratio of 1:1:1. The specificity reflects the ability to separate compounds into comprehensive phytochemical substances with similar terpenes or molecules. The analysis was performed with a standard solution of 1,8-cineole, terpinen-4-ol, and (−)-α-bisabolol to establish the RT compared to a blank solution of the solvent (Supplementary Figure S6) and the sample solution of 1,8-cineole, TTO, and (−)-α-bisabolol. It was found that the RT of all compounds in the standard sample solution and test sample solution are practically the same (Table 6). The chromatograms of the standard sample (Figure 2) and test sample (Figure 3) had a high resolution of main peaks and sufficient NTP values. No blank interference was observed at the RT of 1,8-cineole, terpinen-4-ol, and (−)-α-bisabolol. These results correspond to the requirements of the acceptance criterion for specificity.

#### 2.2.3. Linearity

The linear correlation was established using results of the GC–MS assay that are proportional to the concentration of each phytochemical in the standard sample solution within the limits of the analytical method. A series of standard solutions of 1,8-cineole, terpinen-4-ol, and (−)-α-bisabolol were prepared. The graphic representation was based on a dependence of the analytical signal on the concentration of the substance. The correlation coefficient (R^2^) between the series was at least 0.995 for each phytochemical in substance that provided evidence for the linearity of the method and the possibility of analyzing the novel plant-based substance within chosen concentrations.

Firstly, for 1,8-cineole in the concentration range from 0 to 0.025 weight %, a linear correlation of the response in the GC–MS assay was observed with a correlation coefficient of 0.999 (Supplementary Figure S7A). Secondly, for terpinen-4-ol in the concentration range from 0 to 0.0075 weight %, a linear correlation of the response in the same study was identical and was observed with a correlation coefficient of 0.999 (Supplementary Figure S7B). Finally, for (−)-α-bisabolol in the concentration range from 0 to 0.025 weight %, a linear dependence of the response in the test was identical with a correlation coefficient of 0.999 (Supplementary Figure S7C). The chromatogram of the standard sample of the substance based on 1,8-cineole in concentration 0.005 weight %, tepinen-4-ol in concentration 0.0015 weight %, and (−)-α-bisabolol in concentration 0.005 weight % is shown in Supplementary Figure S8. The raw data are presented in Supplementary Table S1 with linear function for each compound in Supplementary Table S2. In conclusion, the linearity and suitability of this GC–MS assay of the terpene-enriched substance in various concentration ranges is confirmed using this method and these chromatographic conditions.

#### 2.2.4. Accuracy

Accuracy is one of most important chromatographic parameters during the validation process. This parameter helps to assess the deviation of the determination mean value of the results from the values accepted as true. The accuracy of the procedure for the arbitrary determination of 1,8-cineole, terpinen-4-ol, and (−)-α-bisabolol was correctly proved by the method of additives. The range of mass concentrations was from 80% to 120% [76], which was corrected for 100% to standard sample solution. The percent of recovery for all compounds was within the range from 98.6% to 101.6%; the RSD ranged from 0.88% to 1.51% and did not exceed 2.0% according to criteria of acceptance. The coefficient of variation (CV) was varied from 0.79% to 2.28%. The results of accuracy are presented in Table 7. The difference of recovery, dispersion, and RSD of (−)-α-bisabolol could be explained due to high molecular mass and a higher ability of being retained in the chromatographic column [77]. These results correspond to the requirements of the acceptance criterion for accuracy.

#### 2.2.5. Intraday and Interday Precision

The intraday and interday precision was investigated in terms of the precision confirmation in standard laboratory conditions and determination of the influence of different random factors. They were determined by analyzing six replicates of three test sample solutions in different concentrations (0.01, 0.1, and 0.2 weight %) from linearity range on the same day and three consecutive days. Repeatability as a main parameter of precision does not depend on a true value of the measured value. Convergence characterizes the degree of consistency of the results of tests obtained by GC–MS assay of the test solution sample of the novel substance based on 1,8-cineole, TTO-enriched terpinen-4-ol, and (−)-α-bisabolol (Supplementary Figure S9). The percent coefficient of variation of both intraday and interday precision was ≤7.1% During the repeatability of this method, the contents of 1,8-cineole, terpinen-4-ol, and (−)-α-bisabolol were measured. According to the results, the CV for intraday ranged from 0.86% to 2.47%. Similar results were obtained for interday precision in that the CV varied from 1.57% to 2.56%. The results of precision are presented in Table 8. It was observed that the lowest results of intraday and interday precision were achieved at a concentration of 0.1 weight %. It was an optimal concentration from the linearity range. These results mostly correspond to the requirements of the acceptance criterion for precision. However, more replicates are needed for the collection of statistical data.

## 3. Materials and Methods

### 3.1. Chemicals and Plant Materials

The substances for the test sample, such as *M. alternifolia* leaf oil (CAS 68647-73-4) standardized in terpinen-4-ol content, 1,8-cineole (CAS 470-82-6), and (−)-α-bisabolol (CAS 23089-26-1), were procured from Sigma-Aldrich (Sigma Chemical Co., Ltd., St. Louis, MO, USA). For the preparation of a standard sample, terpinen-4-ol (CAS 562-74-3; 20126-76-5), 1,8-cineole (CAS 470-82-6), and (−)-α-bisabolol (CAS 23089-26-1) were procured from Ventos (Ernesto Ventos S.A., Barcelona, Spain). The test sample solution was prepared by mixing the TTO, 1,8-cineole, and (−)-α-bisabolol in a mass ratio of 1:1:1. The characteristics of the used substances are presented in Table 9. The chloroform of technical grade was a solvent for GC–MS analysis.

### 3.2. Standard and Test Sample Solution Preparation

The standard solution sample was prepared by mixing 0.15 mL of terpinen-4-ol standard, 0.5 mL of 1,8-cineole standard, and 0.5 mL of (−)-α-bisabolol standard. The terpinen-4-ol in the amount of 0.15 mL was determined based on the standardized content of terpinen-4-ol of more than 30% in TTO. The standard solution sample was prepared by diluting two hundred times in 10 mL of chloroform to prepare graduated solutions of lower concentrations. Solutions for the research using the method of additives were also prepared from final standard solution. The shelf life of the standard sample was 7 days at a storage temperature of 2 to 8 °C.

The test solution sample was prepared by mixing 0.5 mL of TTO, 0.5 mL of 1,8-cineole, and 0.5 mL of (−)-α-bisabolol in a mass ratio 1:1:1 with 10 mL of chloroform and diluting four hundred times up to a final concentration for further GC–MS analysis. This solution was used freshly prepared before each analysis with optimization based on linearity and accuracy results. Recovery was achieved at different sample concentrations.

### 3.3. GC–MS Conditions

The development and validation of GC–MS analysis was conducted using the gas chromatograph Shimadzu GCMS TQ 8040 system (Shimadzu, Kyoto, Japan) coupled with the mass spectrometer QP-2010 Ultra (Shimadzu; EI source at 230 °C with 70 eV; scanning 29–500 *m*/*z* at 3.3 Hz), an autosampler for static headspace analysis (AOC 5000 plus, Shimadzu, Kyoto, Japan), and a capillary column HP-INNOWAX (30 m × 0.25 mm i.d., 0.25 µm film thickness). The carrier gas was helium of 99.99% purity, flowing at a constant rate of 1.5 mL/min. The temperature graduate program of the column was programmed to have several gradual ramps: (a) from 80 to 140 °C at a rate of 10 °C/min; (b) from 140 to 280 °C at a rate of 20 °C/min; (c) at this point, the GC oven was held at 280 °C for 10 min. The injector heater’s temperature was set to 280 °C. The splitless mode was 1:40 during the analysis. The MS data were obtained by electron impact ionization and used for comparing with the NIST-2017 (National Institute of Standards and Technology, Gaithersburg, MD, USA) and Wiley-08 (Wiley, New York, NY, USA) databases. The MS transfer line and ion source were at 230 °C. The injected volume of sample was 1 µL. The results of phytoconstituent profiles after GC–MS analysis were compared using Adams libraries [44,60,61]. All analyses were carried out in triplicate for statistical data processing.

### 3.4. Validation Study of the Analytical Method

The GC–MS analysis was validated in according with the EU Commission regulation guidelines [73,78,79], the *European Pharmacopoeia* 11.0 [71], the *Russian Pharmacopoeia* (XIV edition) [72], and ICH guidelines [80]. The parameters for validation of this analytical method were suitability of the chromatographic system, specificity, linearity, accuracy, and reproducibility. The calculation of the content of each component was carried out according to the method of internal normalization. The suitability of the selected chromatographic conditions was evaluated using solution of linalyl acetate at 130 °C and confirmed by the determination of NTPs, resolution between peaks, and RSD of peaks. During a specificity test, the test sample solution and standard sample solution were compared by the parameters such as RT, NTPs, peak resolution, and peak symmetry. The influence of solvent or organic impurities from substances is absent. The linearity was confirmed at six concentrations of the 1,8-cineole, terpinen-4-ol and (−)-α-bisabolol from 0 to 0.5 weight % about the normalized value added to blank solution (chloroform) to determine a linear dependence of signal abundance in six replicates. Accuracy was estimated by the method of additions for at least three concentrations of the novel substance within the analytical area of linearity in nine replicates. For assay of this parameter, 1,8-cineole, terpinen-4-ol, and (−)-α-bisabolol were added separately to the test solution containing the components at a concentration of 80%, 100%, and 120% of the initial concentration. Acceptance criteria of accuracy must belong to the range from 98% to 102%. Moreover, intraday and interday precision was determined by the calculation of CV and RSD of the six parallel measurements.

### 3.5. Statistical Analysis

Results of the research are presented as mean ± standard deviation calculated from the parallel replicates for each validation parameter. The statistical analysis was performed by one-way analysis of variance (ANOVA) using Microsoft Excel (version 2016) and by Student’s *t*-test using Statistica soft-ware (version 9.0, StatSoft, Tulsa, OK, USA). Results were considered statistically significant for *p* ≤ 0.05.

## 4. Conclusions

The research revealed the development and validation of an accurate, sensitive, and reproducible GC–MS method for the analysis of the main phytochemicals in an innovative substance based on TTO, 1,8-cineole, and (−)-α-bisabolol at a specific mass ratio for the treatment of SD. At the same time, the proper chromatographic conditions were chosen and validated to obtain high specificity, sensitivity, linearity, accuracy, and precision according to the European and ICH guidelines, the *European Pharmacopoeia* (XI edition), and the *Russian Pharmacopoeia* (XIV edition). This method provides a new approach for the analysis of innovative multicomponent substances of plant origin. Furthermore, validated GC–MS analysis provides a basis for development of the method to control pharmaceutical products with this novel plant-based substance.

## Figures and Tables

**Table 1:** Phytoconstituents of the standard sample.

No.	Compound	MW	Chemical Class	RT (min)	Literature RI [44]	Relative Content, %
1	1,8-cineole	154.25	Bicyclic epoxygenated monoterpene	1.775	1032	42.06
2	m-cymene	134.22	Aromatic monoterpene	2.158	1022	0.31
3	2-hexanol	102.17	Six carbon alcohol	2.434	865	0.48
4	Terpinen-4-ol	154.25	Cyclic oxygenated monoterpene	5.392	1177	25.00
5	endo-borneol	154.25	Cyclic oxygenated monoterpene	6.488	1166	0.19
6	α-bisabolene	204.36	Monocyclic sesquiterpene	6.777	1540	0.07
7	Trans-ascaridole glycol	170.25	Cyclic oxygenated monoterpene	9.773	1273	0.17
8	(−)-α-bisabolol	222.37	Monocyclic sesquiterpene alcohol	10.492	1683	31.70

**Table 2:** Phytoconstituents of the test sample based on TTO, 1,8-cineole:(−)-α-bisabolol in a 1:1:1 mass ratio.

No.	Compound	MW	Chemical Class	RT (min)	Main *m*/*z*	Relative Content, %
1	β-myrcene	136.23	Acyclic monoterpene	1.55	93	0.27
2	α-phellandrene	136.24	Cyclic monoterpene	1.66	93	3.86
3	α-terpinene	136.24	Cyclic monoterpene	1.67	93	3.86
4	D-limonene	136.24	Cyclic monoterpene	1.77	68	0.45
5	1,8-cineole	154.25	Bicyclic epoxygenated monoterpene	1.83	43	25.63
6	γ-terpinene	136.24	Cyclic monoterpene	2.05	93	10.36
7	m-cymene	134.22	Aromatic monoterpene	2.23	119	3.78
8	Terpinen-4-ol	154.25	Cyclic oxygenated monoterpene	5.42	71	16.98
9	α-terpineol	154.25	Cyclic monoterpene	6.49	59	15.31
10	δ-cadinene	204.36	Bicyclic sesquiterpene	7.08	161	0.31
11	β-bisabolol	222.36	Sesquiterpene	10.13	82	0.27
12	β-caryophyllene	204.36	Sesquiterpene	10.42	93	0.79
13	(−)-α-bisabolol	222.37	Monocyclic sesquiterpene alcohol	10.49	119	27.67
14	Farnesol	226.36	Acyclic monoterpene	10.80	69	2.52
15	Trans-geranylgeraniol	290.48	Acyclic monoterpene	11.00	69	0.55

**Table 3:** MS characteristics of major compounds in the novel plant-based substance.

No.	Compound	MW	Chemical Formula	*m*/*z* Peaks	
				Main Peak	Highest Peaks
1	1,8-cineole	154.25	C_10_H_18_O	43	58, 71, 81, 93, 108, 139, 154
2	Terpinen-4-ol	154.25	C_10_H_18_O	71	43, 93, 111, 136, 154
3	(−)-α-bisabolol	222.37	C_15_H_26_O	119	41, 43, 69, 71, 93, 109, 134, 161, 189, 204

**Table 4:** Detailed results of validation of GC–MS method.

Parameters	Typical Acceptance Criteria			Observations	
			1,8-cineole	terpinen-4-ol	(−)-α-bisabolol
System suitability	NTPs ^a^ for each peak ≥ 10,000		19,319	179,154	678,580
	Resolution between all peaks ≥ 1.5		≥2.0	≥2.0	≥2.0
	RSD ^b^ of RT < 1.0%		0.05%	0.04%	0.02%
	RSD ^b^ of each peak of <2.0–3.0%		2.6%	3.2%	2.7%
	Peak skewness factor = 1		1	1	1
Specificity	RT of these phytocompounds	test sample	1.768	5.388	10.492
		standard	1.774	5.389	10.490
	NTPs	test sample	19,275	179,021	678,839
		standard	19,319	179,154	678,580
	Content of compound in the substance		23.01–23.51%	18.57–18.75%	29.74–30.16%
Linearity	Range (n = 3)				
	Square of the correlation coefficient for each substance should be ≥0.99		0.999	0.999	0.999
Accuracy	Recovery (n = 9), %		100.2 ± 2.3	100.7 ± 2.1	100.0 ± 3.5
	Dispersion, %		1.008	0.788	2.280
	Variation coefficient should be ≤2.0%		1.0%	0.9%	1.5%
Reproducibility	Mean content (n = 6), %		0.0132	0.006	0.0253
	Variation coefficient should be ≤2.0%		1.28%	1.96%	1.99%

^a^ The number of theoretical plates (NTPs) characterizes the resolution of phytochemicals during GC–MS analysis; ^b^ relative standard deviation (RSD) shows the spread of values for each analytical parameter during GC–MS analysis.

**Table 5:** Results of test for suitability of the chromatographic system.

Substance	Retention Time		Peak Area		Peak Asymmetry Factor	Average NTPs
	Average Value, min	RSD, %	Average Value	RSD, %		
1,8-cineole	1.774	0.05	3,503,956	2.6	1	19,319
Terpinen-4-ol	5.389	0.04	1,815,200	3.2	1	179,154
(−)-α-bisabolol	10.489	0.02	2,376,829	2.7	1	678,580

**Table 6:** Results of test for specificity of the chromatographic system.

Sample	RT, min		
	1,8-cineole	terpinen-4-ol	(−)-α-bisabolol
Standard sample solution	1.774	5.389	10.490
Test sample solution	1.768	5.388	10.492

**Table 7:** Results of accuracy of the GC–MS assay.

Compound	Average Content, %	Added Content, %	Recovery, %	Mean Recovery, %	CV ^a^, %	RSD, %
1,8-cineole	0.001	0.0002	99.4	100.2	1.01	1.0
	0.005	0.001	100.5			
	0.01	0.002	100.8			
Terpinen-4-ol	0.0003	0.00006	100.0	100.7	0.79	0.88
	0.0015	0.0003	101.1			
	0.003	0.0006	101.2			
(−)-α-bisabolol	0.001	0.0002	98.6	100.0	2.28	1.51
	0.005	0.001	101.6			
	0.01	0.002	99.9			

^a^ The coefficient of variation (CV) is the mathematical ratio of the standard deviation to the mean of analytical parameter.

**Table 8:** Results of tests for precision of the chromatographic system.

	The Test Sample Solution		
Concentration of the Plant-Based Substance, Weight%	Compound	Precision for Each Compound (CV, %)	
		Intraday (n = 6)	Interday (n = 18)
0.02	1,8-cineole	1.38	1.57
	Terpinen-4-ol	1.97	1.82
	(−)-α-bisabolol	2.47	2.19
0.1	1,8-cineole	0.86	1.27
	Terpinen-4-ol	1.16	2.53
	(−)-α-bisabolol	1.92	2.13
0.2	1,8-cineole	1.28	1.64
	Terpinen-4-ol	1.96	2.52
	(−)-α-bisabolol	1.99	2.56

**Table 9:** Substances for preparation of standard and analyzed solution samples.

No.	Chemical	Origin	CAS Number	Assay	Manufacturer
Substances for standard sample					
1	Terpinen-4-ol natural	Leaves of *Eucalyptus globulus*	562-74-3; 20126-76-5	>99.5%	Ernesto Ventos S.A., Spain
2	1,8-cineole (eucalyptol) natural	Leaves of *Eucalyptus spp*.	470-82-6	>99.5%	Ernesto Ventos S.A., Spain
3	(−)-α-bisabolol natural	Leaves of *Vanillomospsis erythropappa*	23089-26-1	>95%	Ernesto Ventos S.A., Spain
Substances for test sample					
4	*Melaleuca alternifolia* leaf oil standardized in terpinen-4-ol content	Leaves of *M. alternifolia*	68647-73-4	>30% of terpinene-4-ol	Bernardi Group, Grasse, France
5	1,8-cineole (eucalyptol)	Leaves of *Eucalyptus spp*.	470-82-6	>99.5%	Wuxi Lous Essence Co., Ltd., Wuxi, Jiangsu, China
6	(−)-α-bisabolol	Leaves of *Hymenocrater yazdianus*	23089-26-1	>95%	Merck KGaA, Darmstadt, Germany

## Data Availability

The data presented in this study are available on request from the corresponding author.

## Supplementary Materials

The following supporting information can be downloaded at https://www.mdpi.com/article/10.3390/antibiotics12101558/s1, Figure S1: GC–MS chromatogram of the standard sample (terpinen-4-ol:1,8-cineole:(−)-α-bisabolol in a 1:1:1 mass ratio); Figure S2: (A) Mass spectrum of terpinen-4-ol in the test sample. (B) Mass spectrum of terpinen-4-ol from the NIST-2017 library; Figure S3: (A) Mass spectrum of 1,8-cineole in the test sample; (B) mass spectrum of 1,8-cineole from the NIST-2017 library; Figure S4: (A) Mass spectrum of (−)-α-bisabolol in the test sample; (B) mass spectrum of (−)-α-bisabolol from the NIST-2017 library; Figure S5: Linalyl acetate chromatogram for total ion current; Figure S6: GC–MS chromatogram of the blank solution of the chloroform in specificity test; Figure S7: The linearity results for the standard solutions of 1,8-cineole (A), terpinen-4-ol (B), (−)-α-bisabolol (C); Figure S8: GC–MS chromatogram of the standard sample of the novel plant-based substance (TTO:1,8-cineole:(−)-α-bisabolol in a 1:1:1 mass ratio) in linearity test; Table S1: Results of linearity of the GC–MS assay for 1,8-cineole, terpinen-4-ol, and (−)-α-bisabolol; Table S2: The linear functions for 1,8-cineole, terpinen-4-ol, and (−)-α-bisabolol standard solutions; Figure S9: GC–MS chromatogram of the test sample solution of the novel plant-based substance (TTO:1,8-cineole:(−)-α-bisabolol in a 1:1:1 mass ratio) in repeatability precision test.
