# Supplementary material for: Development and Validation of a Gas Chromatography–Mass Spectrometry Method for the Analysis of the Novel Plant-Based Substance with Antimicrobial Activity
Source: Antibiotics (Basel). 2023 Oct 22;12(10):1558. doi: 10.3390/antibiotics12101558 (PMC10603869; doi:10.3390/antibiotics12101558)
Supplement: Supplementary file 1 [file antibiotics-12-01558-s001.zip › Supplementary materials.pdf]

Figure S1. GC–-MS chromatogram of the standard sample (erpinene-4-ol:1,8-cineole:(−)-α-bisabolol in a 1:1:1 mass ratio).

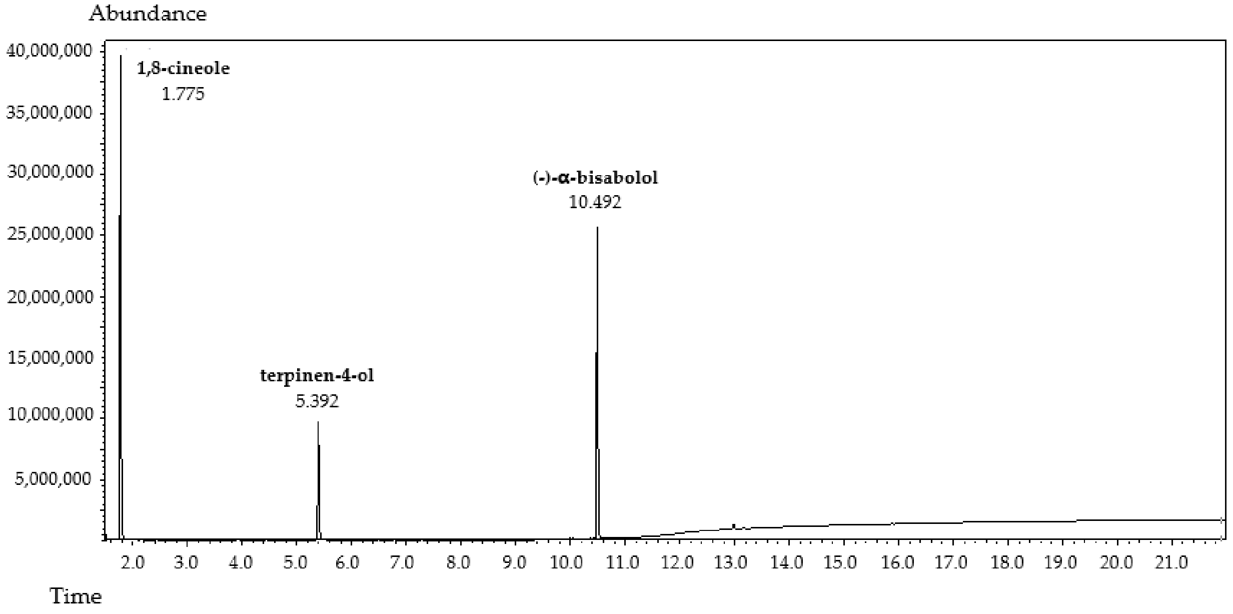

Figure S1. GC-MS chromatogram of the standard sample (terpinen-4-ol:1,8-cineole:(-)-α-bisabolol in a 1:1:1 mass ratio

Figure S2. (A) Mass spectrum of terpinen-4-ol in the test sample. (B) Mass spectrum of terpinen-4-ol from the NIST-2017 library.

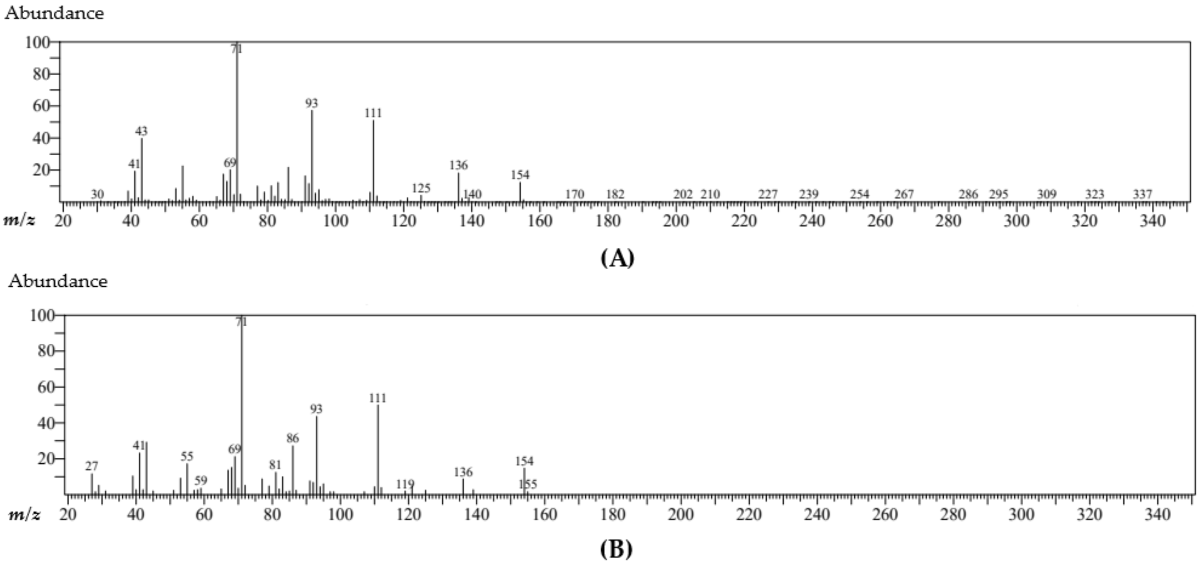

Figure S2. (A) Mass spectrum of terpinen-4-ol in the test sample; **(B)** mass spectrum of terpinen-4-ol from the NIST-2017 library.

Figure S3. (A) Mass spectrum of 1,8-cineole in the test sample; (B) mass spectrum of 1,8-cineole from the NIST-2017 library.

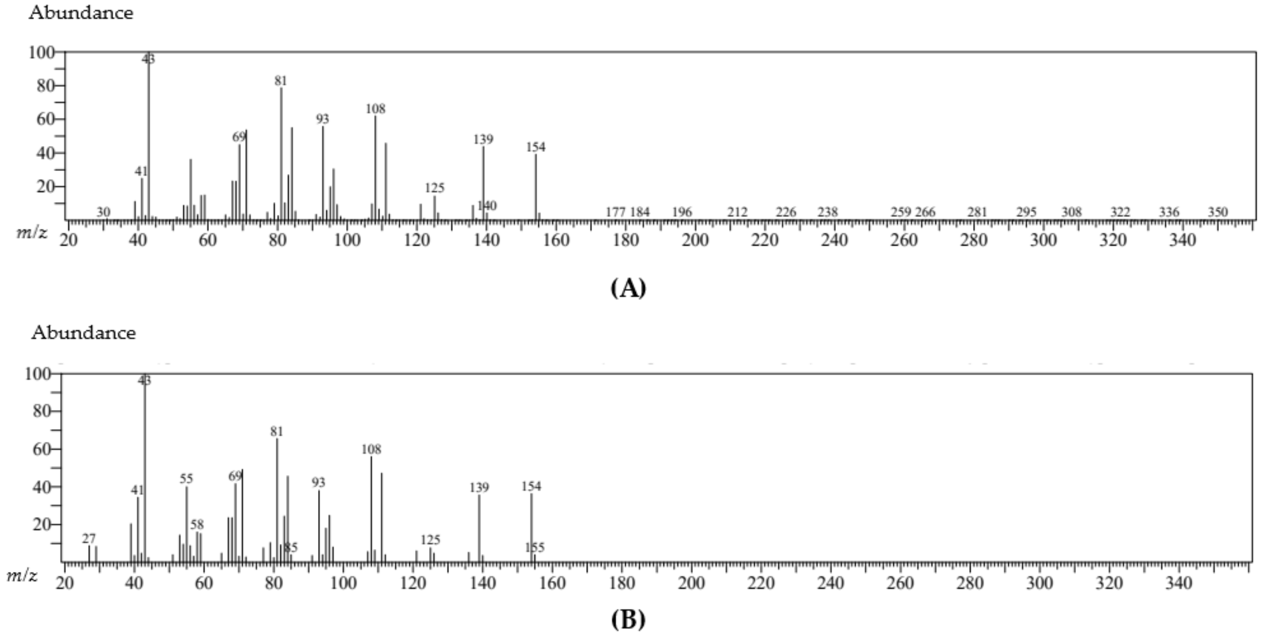

Figure S3. (A) Mass spectrum of 1,8-cineole in the test sample; **(B)** mass spectrum of 1,8-cineole from the NIST-2017 library.

Figure S4. (A) Mass spectrum of (−)-α-bisabolol in the test sample; (B) mass spectrum of (−)-α-bisabolol from the NIST-2017 library.

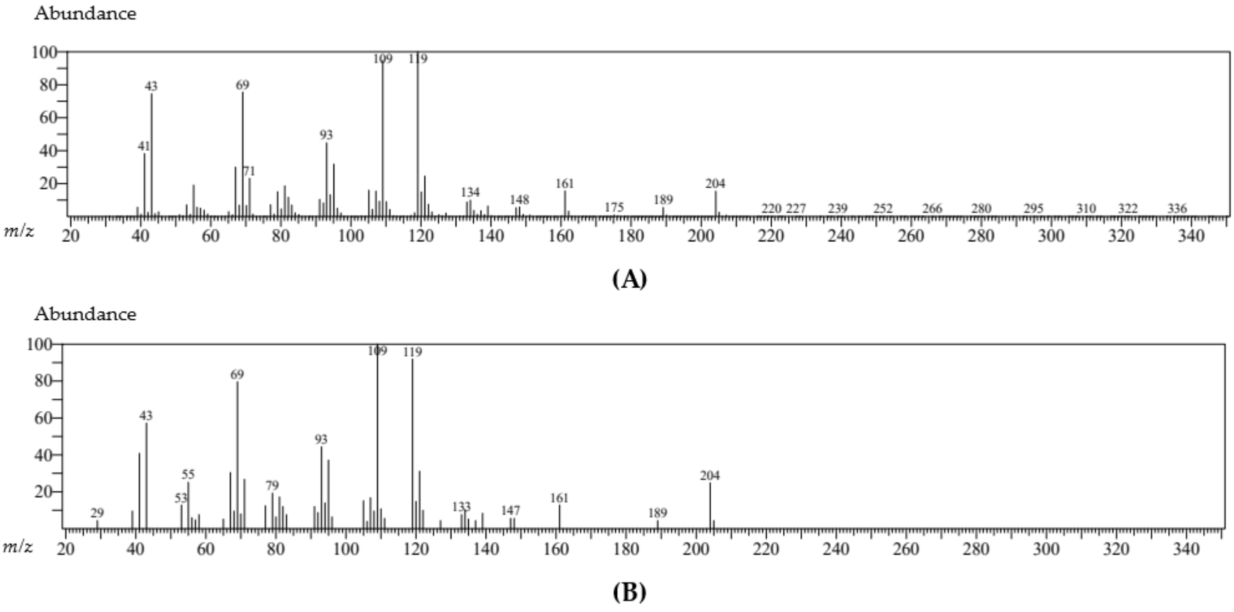

Figure S4. (A) Mass spectrum of (-)-α-bisabolol in the test sample; **(B)** mass spectrum of (-)-α-bisabolol from the NIST-2017 library.

Figure S5. Linalyl acetate chromatogram for total ion current.

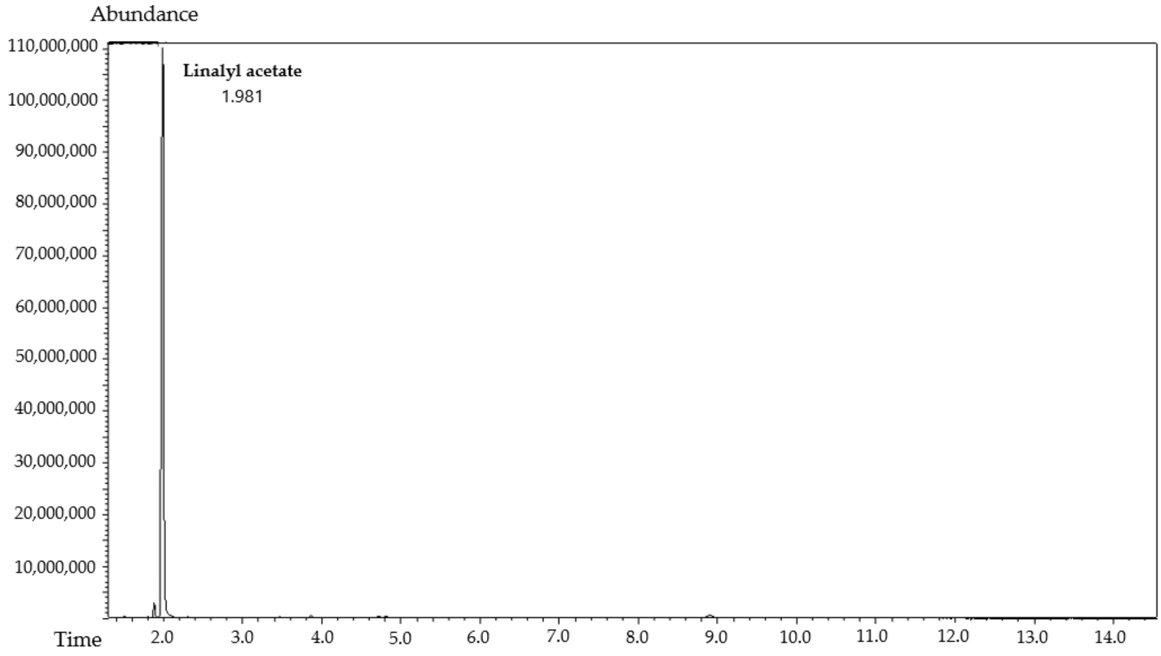

Figure S5. Linalyl acetate chromatogram for total ion current.

Figure S6. GC—-MS chromatogram of the blank solution of the chloroform in specificity test.

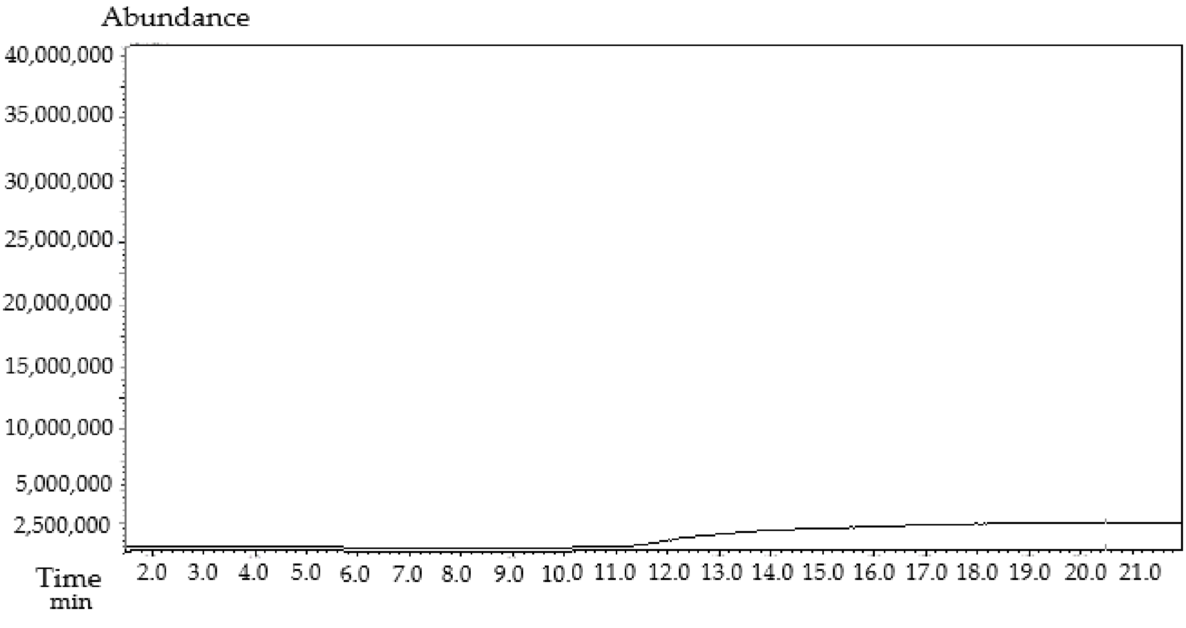

Figure S6. GC–MS chromatogram of the blank solution of the chloroform in specificity test.

Figure S7. The linearity results for the standard solutions of 1,8-cineole (A), terpinen-4-ol (B), (−)-*α*-bisabolol (C).

Figure S7. Linearity results for the standard solutions of 1,8-cineole **(A)**, terpinen-4-ol **(B)**, and (-)-α-bisabolol **(C)**.

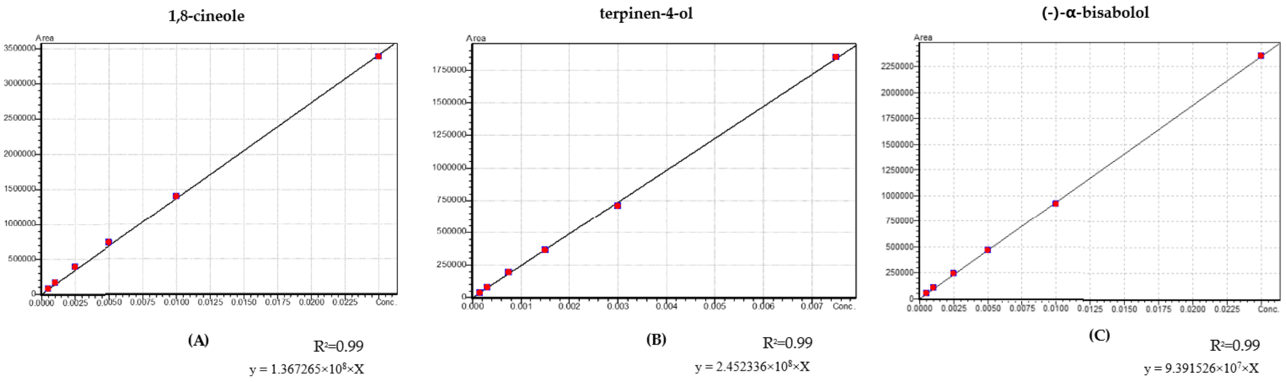

Figure S8. GC—MS chromatogram of the standard sample of the novel plant-based substance (TTO:1,8-cineole:(−)-*α*-bisabolol in a 1:1:1 mass ratio) in linearity test.

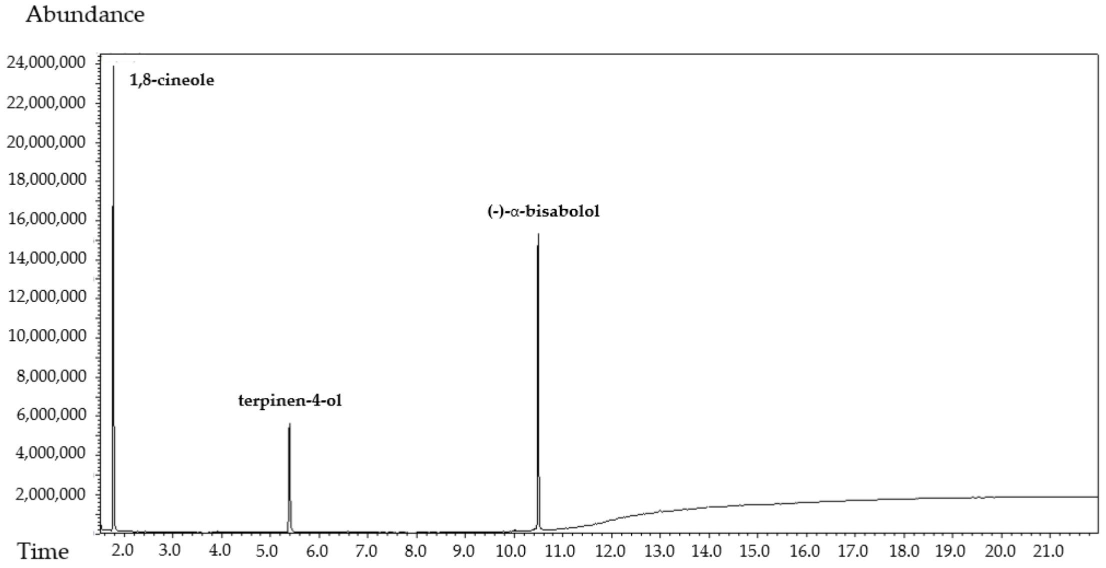

Figure S8. GC-MS chromatogram of the standard sample of the novel plant-based substance (TTO:1,8-cineole;(-)-α-bisabolol in a 1:1:1 mass ratio) in linearity test.

Table S1. Results of linearity of the GC–MS assay for 1,8-cineole, terpinen-4-ol, and (-)-*α*-bisabolol.

Content from standardized value, %	Concentration of the standard solution of 1,8-cineole, weight %	Concentration of the standard solution of terpinen-4-ol, weight %	Concentration of the standard solution of (-)-*α*-bisabolol, weight %	Analytical response for each component (peak area)
0<br>(Blank)	0	0	0	0<br>0<br>0
0.01	0.0005	0.00015	0.0005	76950<br>38594<br>56910
0.02	0.001	0.0003	0.001	160758<br>79454<br>109763
0.05	0.0025	0.00075	0.0025	388469

				194984<br>247406
0.1	0.005	0.0015	0.005	731154<br>361610<br>466716
0.2	0.01	0.003	0.01	1397723<br>708694<br>924232
0.5	0.025	0.0075	0.025	3390676<br>1849925<br>2352327

Table S2. The linear functions for 1,8-cineole, terpinen-4-ol, and (-)-α-bisabolol standard solutions.

Compound	R^2	Linear function
1,8-cineole	0.999	$Y = 1.367265 \times 10^8 \times X$
Terpinen-4-ol	0.999	$Y = 2.452336 \times 10^8 \times X$
(-)-α-bisabolol	0.999	$Y = 9.391526 \times 10^7 \times X$

Figure S9. GC–MS chromatogram of the test sample solution of the novel plant-based substance (TTO:1,8-cineole:(−)-α-bisabolol in a 1:1:1 mass ratio) in repeatability precision test.

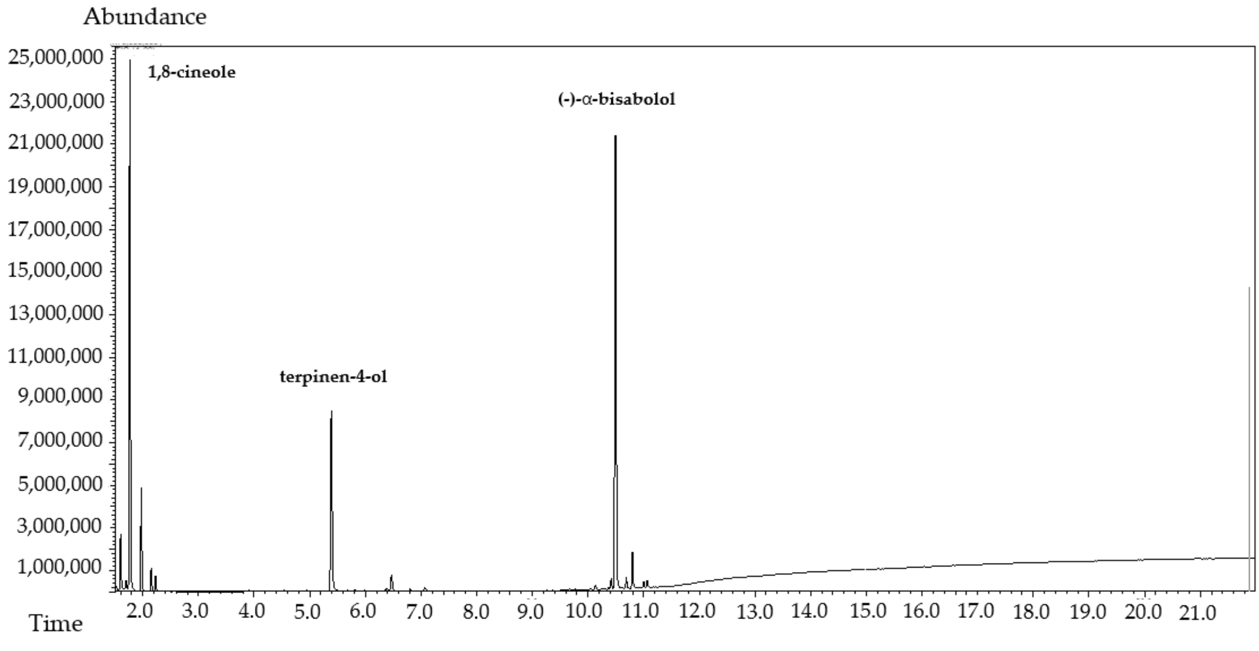

Figure S9. GC-MS chromatogram of the test sample solution of the novel plant-based substance (TTO:1,8-cineole:(-)-α-bisabolol in a 1:1:1 mass ratio) in precision test.
