# Supplementary material for: Development and Validation of a Gas Chromatography–Mass Spectrometry Method for the Analysis of the Novel Plant-Based Substance with Antimicrobial Activity
Source: Antibiotics (Basel). 2023 Oct 22;12(10):1558. doi: 10.3390/antibiotics12101558 (PMC10603869; doi:10.3390/antibiotics12101558)
Supplement: Supplementary file 1 [file antibiotics-12-01558-s001.zip › Supplementary Tables S1 and S2.pdf]

Table S1. Results of linearity of the GC–MS assay for 1,8-cineole, terpinen-4-ol, and (-)-α-bisabolol.

Content from standardized value, %	Concentration of the standard solution of 1,8-cineole, weight %	Concentration of the standard solution of terpinen-4-ol, weight %	Concentration of the standard solution of (-)-α-bisabolol, weight %	Analytical response for each component (peak area)
0<br>(Blank)	0	0	0	0<br>0<br>0
0.01	0.0005	0.00015	0.0005	76950<br>38594<br>56910
0.02	0.001	0.0003	0.001	160758<br>79454<br>109763
0.05	0.0025	0.00075	0.0025	388469<br>194984<br>247406
0.1	0.005	0.0015	0.005	731154<br>361610<br>466716
0.2	0.01	0.003	0.01	1397723<br>708694<br>924232
0.5	0.025	0.0075	0.025	3390676<br>1849925<br>2352327

Table S2. The linear functions for 1,8-cineole, terpinen-4-ol, and (-)-α-bisabolol standard solutions.

Compound	R^2	Linear function
1,8-cineole	0.999	$Y = 1.367265 \times 10^8 \times X$
Terpinen-4-ol	0.999	$Y = 2.452336 \times 10^8 \times X$
(-)-α-bisabolol	0.999	$Y = 9.391526 \times 10^7 \times X$
