# Supplementary figures and images for: Development and Validation of a Gas Chromatography–Mass Spectrometry Method for the Analysis of the Novel Plant-Based Substance with Antimicrobial Activity
Source: Antibiotics (Basel). 2023 Oct 22;12(10):1558. doi: 10.3390/antibiotics12101558 (PMC10603869; doi:10.3390/antibiotics12101558)

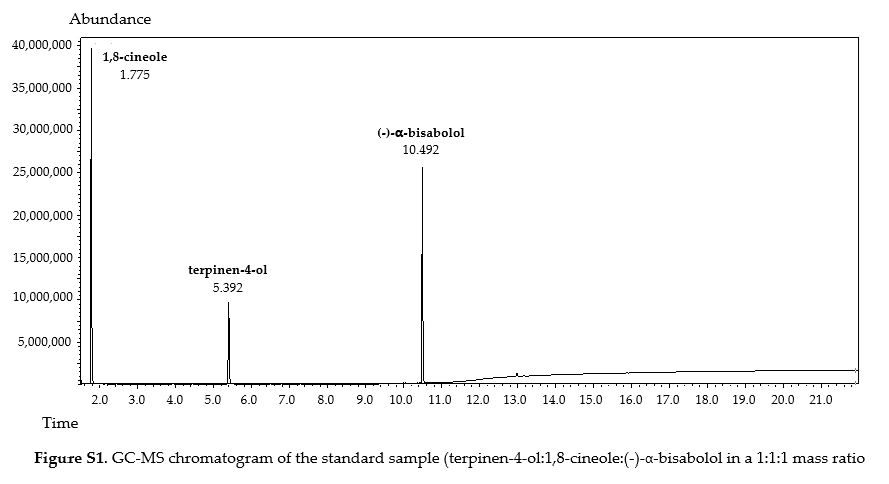

Supplement: Supplementary file 1 [file antibiotics-12-01558-s001.zip › Supplemetary Figure S1.png]

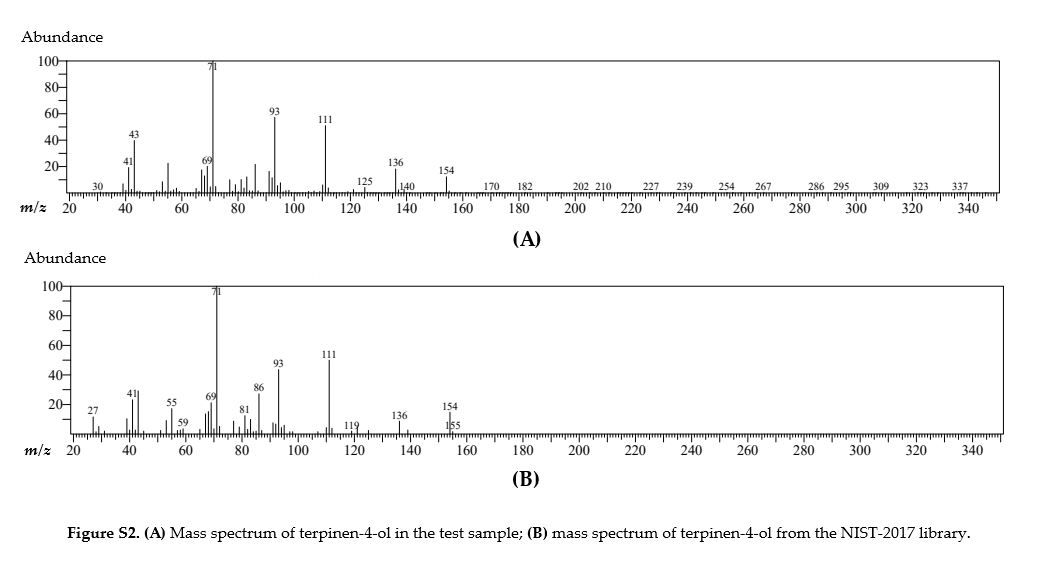

Supplement: Supplementary file 1 [file antibiotics-12-01558-s001.zip › Supplementary Figure S2.png]

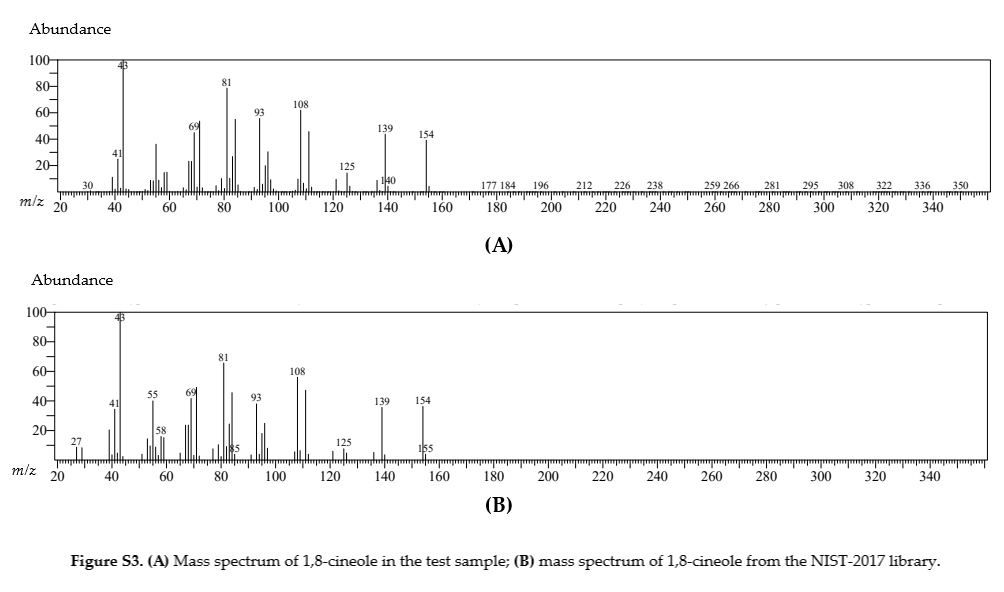

Supplement: Supplementary file 1 [file antibiotics-12-01558-s001.zip › Supplementary Figure S3.png]

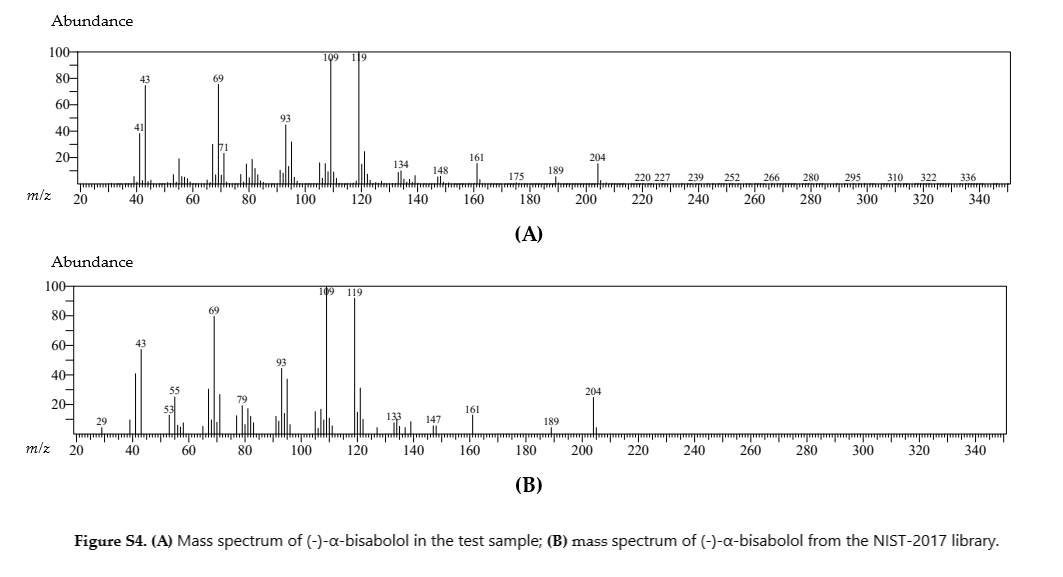

Supplement: Supplementary file 1 [file antibiotics-12-01558-s001.zip › Supplementary Figure S4.png]

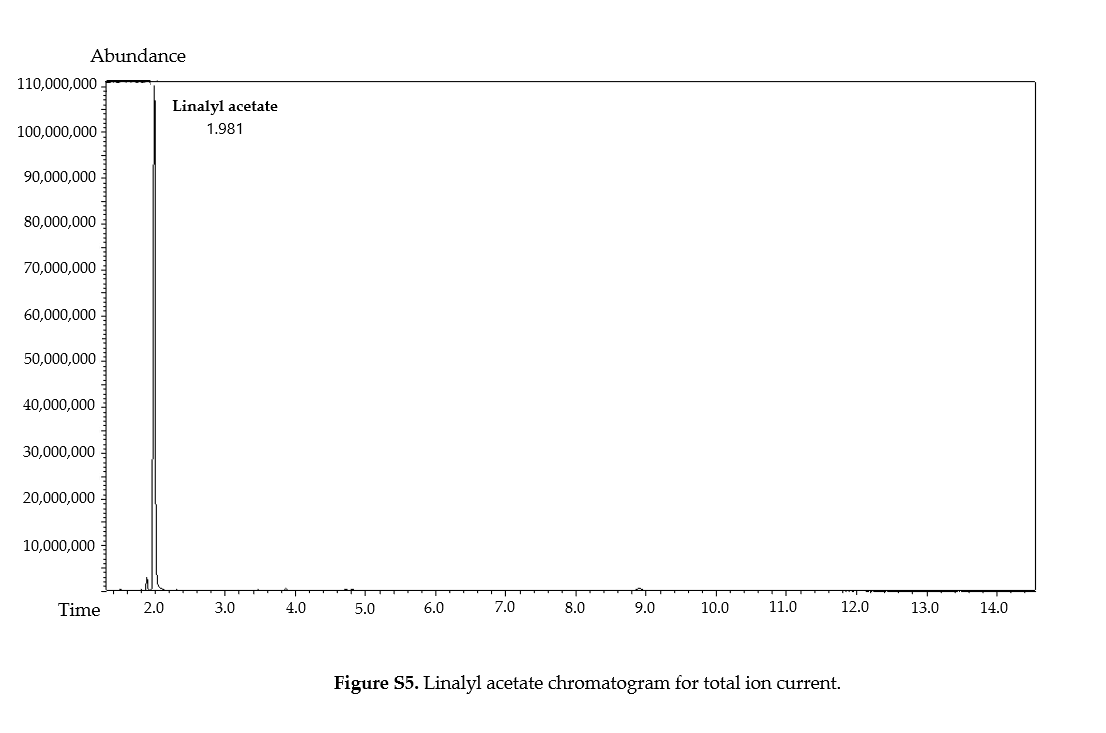

Supplement: Supplementary file 1 [file antibiotics-12-01558-s001.zip › Supplementary Figure S5.png]

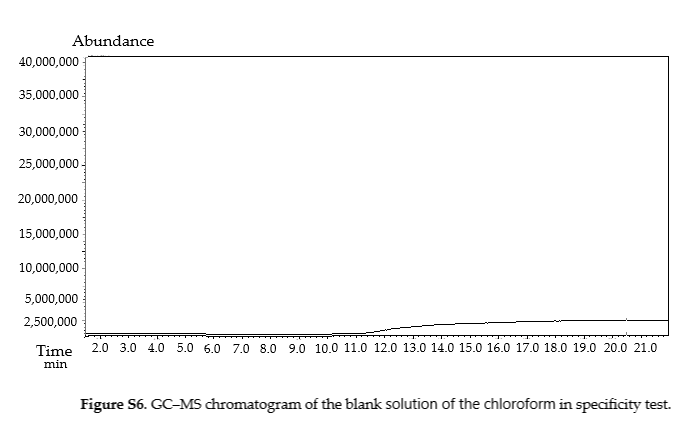

Supplement: Supplementary file 1 [file antibiotics-12-01558-s001.zip › Supplementary Figure S6.png]

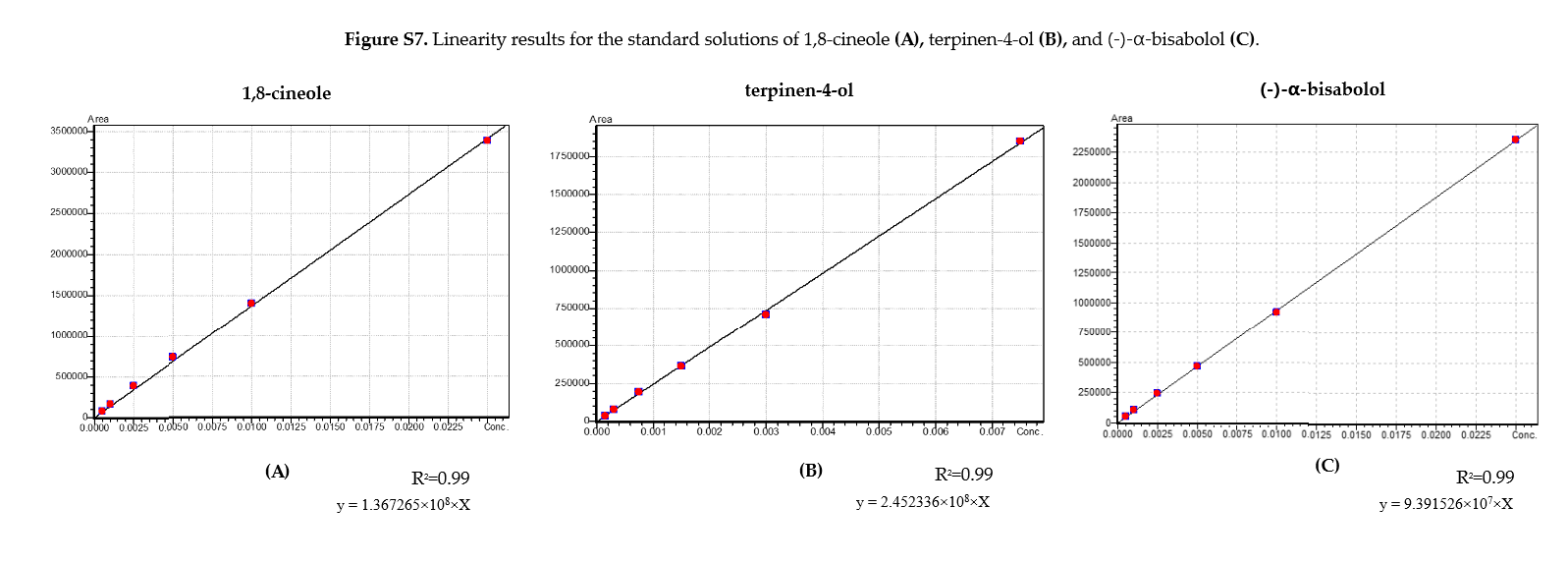

Supplement: Supplementary file 1 [file antibiotics-12-01558-s001.zip › Supplementary Figure S7.png]

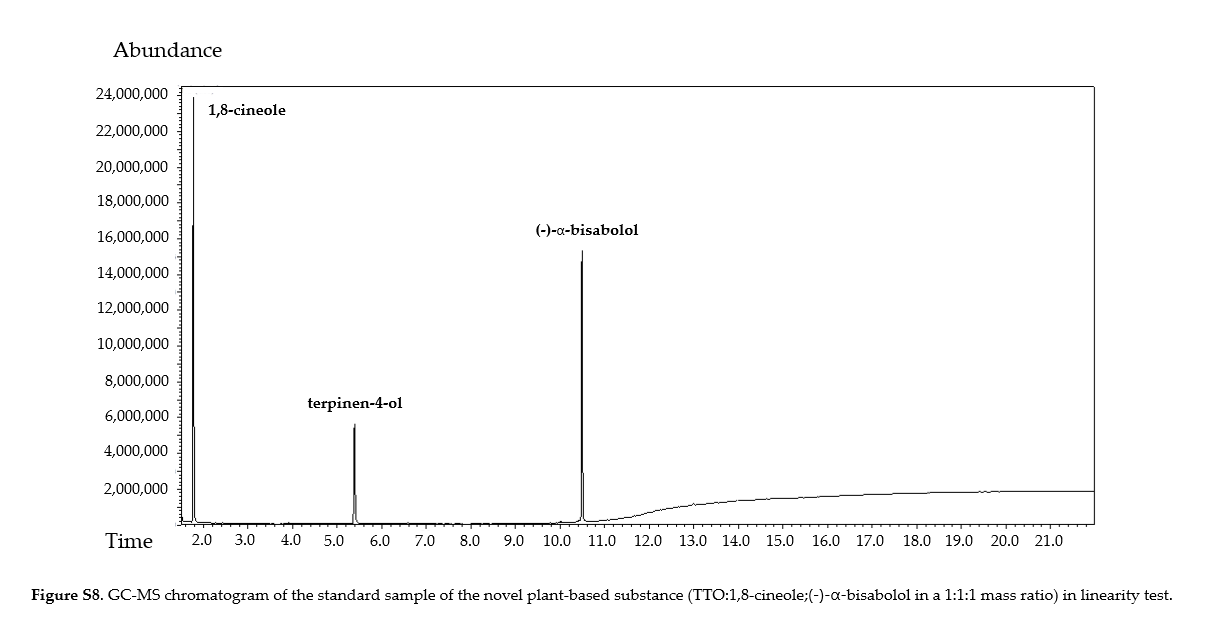

Supplement: Supplementary file 1 [file antibiotics-12-01558-s001.zip › Supplementary Figure S8.png]

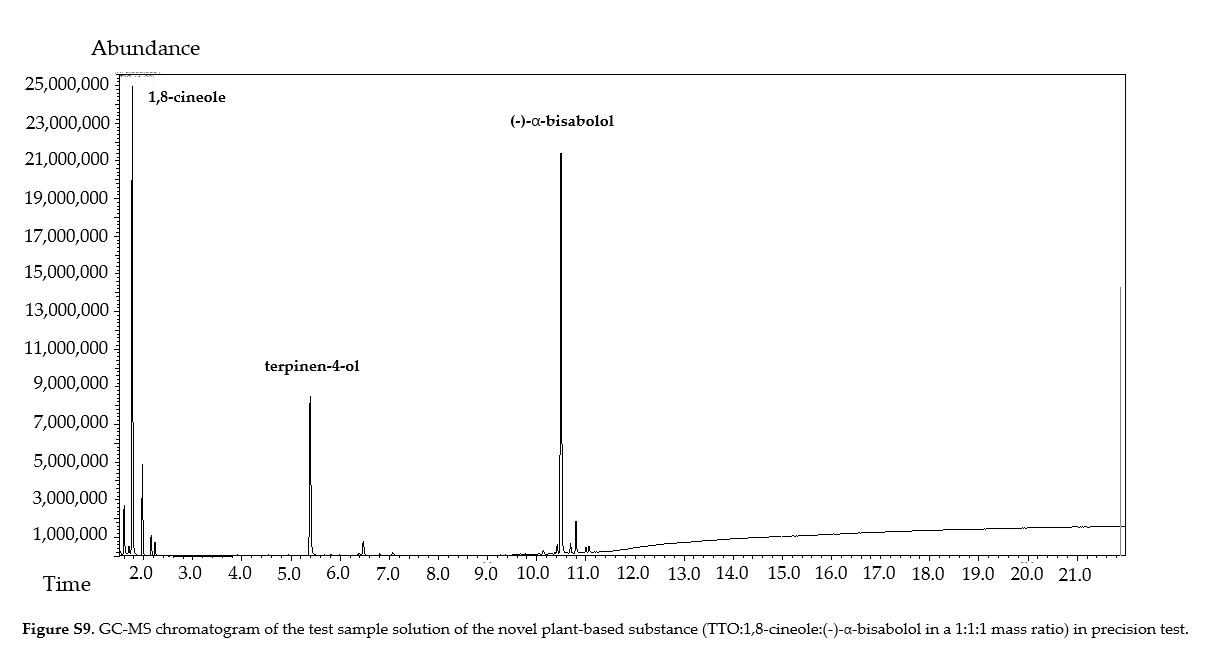

Supplement: Supplementary file 1 [file antibiotics-12-01558-s001.zip › Supplementary Figure S9.png]
